# Beyond Human Vision: Revolutionizing the Localization of Diminutive Sessile Polyps in Colonoscopy

**DOI:** 10.3390/bioengineering12111234

**Published:** 2025-11-11

**Authors:** Mahsa Dehghan Manshadi, M. Soltani

**Affiliations:** 1 Department of Mechanical Engineering, K. N. Toosi University of Technology, Tehran 1999143344, Iran; mahsadehghan@email.kntu.ac.ir; 2 Cancer Institute of Iran, Tehran University of Medical Sciences (TUMS), Tehran 1416753955, Iran; 3 Department of Electrical and Computer Engineering, University of Waterloo, Waterloo, ON N2L 3G1, Canada; 4 Centre for Biotechnology and Bioengineering (CBB), University of Waterloo, Waterloo, ON N2L 3G1, Canada

**Keywords:** gastrointestinal disorders, artificial intelligence, polyp detection, YOLO-V8, NBI, colorectal cancer

## Abstract

Gastrointestinal disorders, such as colorectal cancer (CRC), pose a substantial health burden worldwide, showing increased incidence rates across different age groups. Detecting and removing polyps promptly, recognized as CRC precursors, are crucial for prevention. While traditional colonoscopy works well, it is vulnerable to specialist errors. This study suggests an AI-based diminutive sessile polyp localization assistant utilizing the YOLO-V8 family. Comprehensive evaluations were conducted using a diverse dataset that was assembled from various available datasets to support our investigation. The final dataset contains images obtained using two imaging methods: white light endoscopy (WLE) and narrow-band imaging (NBI). The research demonstrated remarkable results, boasting a precision of 96.4%, recall of 93.89%, and F1-score of 94.46%. This success can be credited to a meticulously balanced combination of hyperparameters and the specific attributes of the comprehensive dataset designed for the colorectal polyp localization in colonoscopy images. Also, it was proved that the dataset selection was acceptable by analyzing the polyp sizes and their coordinates using a special matrix. This study brings forth significant insights for augmenting the detection of diminutive sessile colorectal polyps, thereby advancing technology-driven colorectal cancer diagnosis in offline scenarios. This is particularly beneficial for gastroenterologists analyzing endoscopy capsule images to detect gastrointestinal polyps.

## 1. Introduction

**Figure 1:**
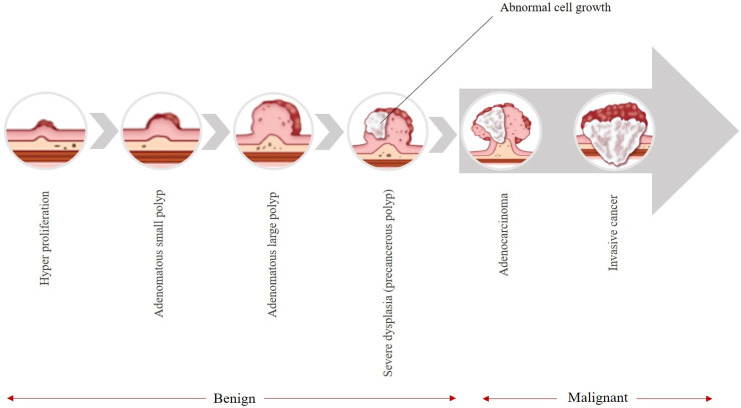
The progression of CRC.

Colorectal cancer (CRC) stands as the third leading cause of cancer-related fatalities, with a mortality rate nearing 51% [1]. CRC stands out as a leading contributor to cancer-related fatalities globally [2]. As it is shown in Figure 1, these types of cancers typically begin as avascular growths known as polyps, which have the potential to become cancerous over time [3]. These colon polyps are classified based on size in Table 1. Polyps that are 5 mm or less in size are classified as diminutive polyps, which are benign, and their cancer risk is low [4]. Polyps between 6 and 9 mm are classified as small and are also benign. The chance of being malignant in polyps 1 cm or more in size is higher than for the two other types [5]. Figure 1 illustrates the natural history of colorectal cancer (CRC), depicting the adenoma–carcinoma sequence. The progression begins with normal colonic mucosa, followed by the formation of benign adenomatous polyps (including diminutive and small sessile types), which may gradually acquire dysplastic changes over 5–10 years. If undetected or untreated, a subset of these lesions can progress to invasive carcinoma and eventually metastatic disease. This figure underscores the critical window for early intervention via colonoscopy.

**Figure 2:**
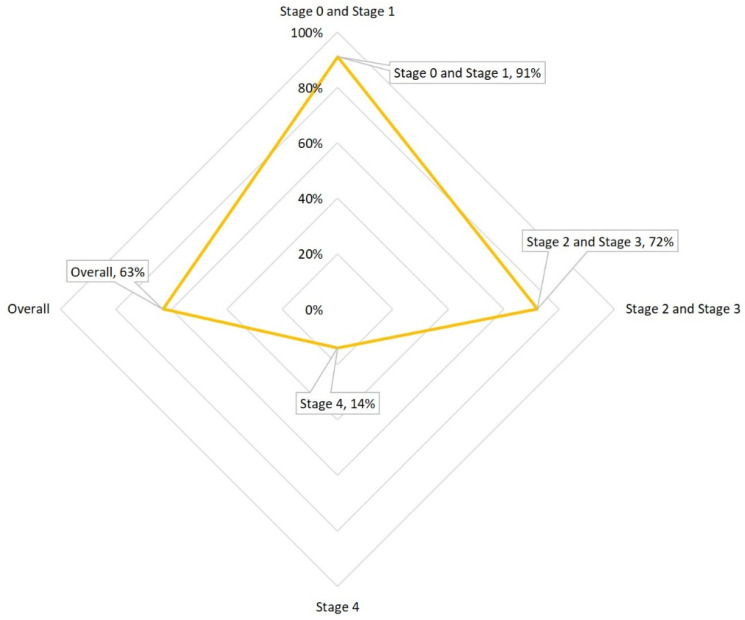
The incidence and survival rates of CRC.

According to [6], the likelihood of cancer arising in polyps under 5 mm is considered very low. In a study examining 15,906 polyps, 4.3% of those exceeding 30 mm were found to be cancerous [7], whereas no cancer was observed in polyps smaller than 5 mm [8]. The National Cancer Institute reports that the likelihood of colorectal cancer occurrence escalates with the colon polyp’s size, noting that 30–50% of polyps greater than 2 cm are malignant. Hence, detecting and removing these adenomas early through colonoscopy is vital in preventing the development of intermediate-stage cancers and it is pivotal for mitigating the incidence and survival rates of CRC (Figure 2). Figure 2 presents global epidemiological trends in colorectal cancer, showing age-standardized incidence rates (per 100,000 population) alongside 5-year relative survival rates across multiple regions. The data highlight a concerning rise in early-onset CRC (diagnosed before age 50) and persistent disparities in survival outcomes, particularly in low-resource settings where screening access is limited. This reinforces the urgency of improving early detection tools, especially for subtle lesions like diminutive sessile polyps.

Table 2 illustrates the various categories of colon polyps and their associated risk of malignancy. The most common type of colon polyp is the adenoma. So, the detection of this type is crucial in colonoscopy. The adenoma detection rate (ADR) plays a key role, but it varies widely (7–53%) due to differences in endoscopists’ skills. Identifying and removing early-stage lesions such as colorectal polyps, particularly diminutive polyps, through colonoscopy is pivotal for mitigating the incidence and mortality rates of CRC [9,10].

**Table 1:** Classify colon polyps based on size [4,5,11].

Size of Colorectal Polyp	Diameter	Cancer Potential
Diminutive	5 mm or less	As noted in a 2020 article, the majority of polyps, 75%, are diminutive and low-risk.
Small	Between 6 and 9 mm	A 2017 study reported that none of the 6523 small polyps had cancer (low risk).
Large	10 mm or more	Larger colon polyps may have a higher risk of cancer. Gastrologists recommend earlier follow-up for polyps greater than 1 cm compared to smaller polyps.

**Table 2:** Types of colon polyps and their cancer risk [10].

Specification	Cancer Potential	More Explanation
Inflammatory	This category could accompany inflammatory bowel disease.	These polyps are generally non-cancerous, although having this disease does elevate the probability of colorectal cancer.
Hamartomatous	This category can develop in individuals with specific genetic conditions such as juvenile polyposis, Peutz–Jeghers syndrome, or Cowden syndrome.	This has a low likelihood of developing cancer (individuals with polyposis syndrome may increase the risk of colorectal cancer in polyps).
Hyperplastic	These polyps are frequently found in the distal colon and are considered common.	These polyps located on the right part of the colon could carry cancer risk. If these are not associated with this situation, the overall cancer risk is typically low.
Adenomas	These polyps account for approximately two-thirds of colon polyps.	These are precancerous.
Sessile–serrated and traditional serrated	These polyps are named for their irregular or rough borders.	These polyps are precancerous.

Colon polyps grow slowly, and they can appear flat, depressed, sessile (without a stalk), or pedunculated (on a stalk resembling a mushroom). Both types of polyps—sessile and pedunculated—have the potential to develop into noncancerous, precancerous, or cancerous forms [10].

Adenomas, comprising approximately 66% of polyps, are precancerous growths that exhibit distinct patterns. Tubular adenomas are smaller, tubular-shaped growths measuring under 0.5 inch (approximately 12.7 mm). Villous adenomas have a greater dimension, with an irregular, cauliflower-like appearance. Both characteristics are found in tubulovillous adenomas. The growth pattern of adenomas could influence the frequency of necessary colonoscopies. Typically, adenomas take about 10 years to develop into cancer, although this timeline may be accelerated in individuals with hereditary syndromes [10].

As outlined by the American Society for Gastrointestinal Endoscopy [12], the standard practice involves removing any polyps detected during screening. Typically, doctors perform colonoscopy to remove polyps, often during the same procedure used for screening. Colonoscopy effectively removes found polyps during screening [13], and this intervention can eliminate the risk of subsequent cancer development. If left untreated, polyps may lead to bleeding, bowel obstruction [14], or potential cancerous growth.

Colonoscopy, an invasive technique using a camera in the digestive tract, is considered the benchmark for identifying polyps and mitigating CRC occurrence and mortality. Studies, such as the one by A.G. Zauber et al. [15], show a significant 53% reduction in mortality through early polyp detection. Despite promising results, colonoscopy is prone to human errors, showcasing a 15–30% rate of polyp detection failures in consecutive procedures, depending on polyp size. Improving technical proficiency and addressing these challenges are essential for enhancing the reliability of this critical screening method.

The complex anatomical structure of the colon and rectum requires advanced expertise for localization and treatment procedures. Furthermore, the constant organ deformations in the colon make it difficult to accurately localize and remove polyps, hindering the ability to track lesion boundaries and complicating complete resection. This intricacy is often subjective and reliant on the experience of the endoscopists [16].

To address these challenges, computer-assisted systems play a pivotal role in minimizing operator subjectivity and enhancing ADR [17]. Moreover, computer-aided techniques for detecting and segmenting images are beneficial for localizing polyps and directing subsequent follow-up procedures, like polypectomy. These methods display accurate polyp locations and margins, aiding in precise surgical interventions [18,19]. For such systems to be viable in clinical settings, they must exhibit performance and algorithmic robustness. By meeting these requirements, computer-assisted systems can significantly contribute to improving the accuracy and efficiency of colorectal polyp detection and treatment processes in clinical practice [20].

Recent strides in computer-aided techniques, particularly the application of one of the most favorable and famous CNN-based methods (the 8th generation of you look only once (YOLO-V8) [21]), have significantly improved the offline detection and diagnosis of colorectal diseases during colonoscopy [22,23]. However, the effectiveness of these CNN-based methods is intricately tied to the availability of annotated image data for training, presenting a notable challenge [24]. Hence, there is a vital need to tackle the challenge of decreasing overlooked adenomas through efficient strategies to standardize the excellence of colonoscopy, emerging as a critical focus in endeavors to prevent CRC.

Several efforts have been made to collect and curate datasets specific to GI conditions, encompassing various polyps. Notably, publicly available datasets exhibit a limitation in that they often originate from a single center or represent a specific population cohort. Public datasets that are standard in research applications often include frames that are sampled from video sequences and primarily include images from one modality. In colonoscopy procedures, white-light endoscopy (WLE) serves as the standard approach [25]. However, experts frequently utilize narrow-band imaging (NBI) [26], a technique categorized as virtual chromo-endoscopy, for enhanced polyp identification [27,28].

Moving forward, it is essential to address these limitations by broadening datasets to include a range of patient profiles, imaging techniques, and endoscope variations. Integrating real-world complexities into the datasets will enhance the development of machine learning models that can robustly handle the challenges encountered in actual clinical scenarios, contributing to the advancement of accurate and effective diagnostic and characterization tools for GI conditions, particularly in the realm of colorectal polyps.

### 1.1. Main Contributions

By addressing key challenges, this paper makes a noteworthy contribution to the field of diminutive sessile colorectal polyp localization by introducing a novel approach of artificial intelligence, particularly through the comparison of different object detection-based YOLO-V8 versions [29] and find the highest accuracy. By tackling the critical challenge of optimizing the tradeoff in both accuracy and efficiency, this research provides clinicians with an AI-based assistant to reduce missed diagnoses, facilitate early detection, and contribute to the prevention of CRC through the localization of precancerous colorectal polyps. The YOLO-V8 family (YOLOV8 n, s, m) has been subjected to rigorous evaluation through detailed testing across three different datasets [30,31,32]. The reason for selecting these three datasets from numerous available datasets is mentioned in the Methodology section. The results, with impressive classification evaluation metrics, highlight the effectiveness of this method.

This research extends its influence beyond immediate implications, laying a foundational basis for further inquiries into diminutive sessile colorectal polyp detection and categorization. Given the significantly evolving landscape of computer vision, establishing a standard dataset becomes paramount. The significant hastening of the advancement of computer-aided diagnosis (CAD) for CRC is anticipated from this work. It is expected that this research will greatly accelerate the development of CAD for CRC. As we navigate the complexities of medical image analysis, this work serves as a major reference point, offering crucial insights and innovative techniques that can guide and drive subsequent research toward more efficient and effective polyp detection assistant systems.

### 1.2. Work Outline

The subsequent sections of this paper are outlined as follows: Section 2 provides an overview of related works. The methodology and its subsections are detailed in Section 3, the results are discussed in Section 4, Section 5 discusses limitations and future prospects, and Section 6 is the Discussion section.

## 2. Related Works

Several studies have focused on improving polyp detection in colonoscopies using convolutional neural networks (CNNs). For instance, the work in [33] introduced a three-dimensional fully convolutional network (3D-FCN) segmentation model that excels in capturing volumetric features for precise boundary delineation, achieving state-of-the-art (SOTA) performance with an F1-score of 0.92 and F2 score of 0.94 on benchmark datasets like CVC-ClinicDB; however, its computational intensity limits real-time applicability, with inference times exceeding 500 ms per frame on standard GPUs, and it underperforms on highly diverse, unseen datasets due to overfitting risks.

Similarly, ref. [34] proposed an innovative fully convolutional network (FCN) structure combining binary classification with a U-Net-like CNN architecture that effectively balances global context and local details to reduce false negatives, demonstrating superior sensitivity (0.95) and specificity (0.96) on the Kvasir-SEG and CVC-ClinicDB datasets and improving accuracy in clinical settings; yet, it requires extensive preprocessing for varying image resolutions, and generalization drops to 0.88 sensitivity on cross-dataset tests like ETIS-Larib, highlighting sensitivity to domain shifts.

Despite significant advances in CNNs for polyp detection, challenges persist, including overfitting and accurate boundary capture. Recent efforts address these through PraNet [35], which employs deep supervision and a reverse attention framework for real-time segmentation that enhances localization efficiency (45 FPS on mid-range GPUs) and generalizability across five datasets, achieving a Dice score of 0.894 and IoU of 0.830 on Kvasir-SEG; nevertheless, it struggles with multi-class segmentation (e.g., distinguishing polyp subtypes), yielding a 5–7% Dice drop in complex scenes with artifacts.

PraNet’s modules have been extended in AMNet [36], a multiscale attention network that boosts edge detection with superior handling of varying polyp shapes and sizes via Res2Net-inspired fusion, attaining Dice scores up to 0.91 on CVC-ClinicDB; that said, its increased model complexity (35 M parameters) raises training time by 20–30% without proportional gains in small-polyp detection (IoU ~0.75).

FANet [37] incorporates feedback attention to refine predictions from coarse representations, attaining a Dice score of 0.92 and IoU of 0.86 on combined datasets and improving small-object accuracy by 4–6% over baselines through iterative refinement; however, it incurs higher inference latency (30–40 ms/frame) and vulnerability to noise in unseen data (e.g., 3% IoU degradation on ETIS-Larib).

Ensemble techniques, particularly dual-model strategies, have gained prominence for polyp segmentation. The dual encoder–decoder in [38] yields promising Dice (0.85) and IoU (0.78) results by reducing redundancy through sequential fusion to enhance boundary precision; however, cumulative latency (60+ ms/frame) hinders real-time use, and it underperforms on imbalanced datasets (recall: ~0.80).

In [39], DDANet employed a dual-decoder network producing grayscale and mask outputs that aids interpretability and surpasses single-decoder baselines by 5% in precision (0.858), with a Dice score of 0.787 and mIoU of 0.701 on Kvasir-SEG; despite this innovation, its sequential implementation limits speed (45 FPS max), and generalization falters on unseen data (Dice drop to 0.75), as later studies show substantial enhancements.

Target localization algorithms leveraging YOLO variants optimize polyp detection. Guo et al. [40] combined YOLO-V3 with active learning that minimizes labeling needs and boosts efficiency in dynamic videos, reducing false positives to 1.5% on colonoscopy videos (precision: 0.92; recall: 0.89); yet, it relies on iterative human feedback, increasing deployment complexity, and the F1-score dips to 0.85 on static images.

Gao et al. [41] integrated feature extraction and fusion into YOLO-V3, excelling in small-polyp detection with an mAP of 0.88 on ETIS-Larib and outperforming vanilla YOLO-V3 by 10% in small-object recall through multi-level fusion that captures fine details; however, its higher parameter count (45 M) elevates memory use, and it falters in low-contrast scenes (precision: ~0.82).

Pacal et al. [42] proposed a real-time YOLO-V4 approach that enables efficient processing on edge devices via CSPNet integration, achieving precision of 0.95, recall of 0.91, and F1 of 0.93 on CVC-ClinicDB to surpass traditional methods in speed (60 FPS); nevertheless, sensitivity to dataset bias yields a 5% recall drop on cross-validation (e.g., Kvasir-SEG).

Advanced YOLO architectures like YOLO-V8 in Ribeiro et al. [43] were evaluated on SUN and PICCOLO, yielding an mAP of 0.94 and recall 0.92 with robust handling of large-scale data and exceptional small-polyp success through scaled variants; however, overfitting on novel datasets requires heavy augmentation, inflating training time by 15%.

Qian et al. [44] fused GAN with YOLO-V4 for robust localization that mitigates data scarcity and enhances generalization via adversarial augmentation, surpassing U-Net with an mAP of 0.91 and precision of 0.93 on combined benchmarks; that said, training instability from adversarial components increases convergence time (2× baseline).

Carrinho [45] explored YOLO-V4 quantization across precision levels, attaining an mAP of 0.829 (Etis-Larib) and 0.910 (CVC-ClinicDB) at INT8 with up to 2× speedup in inference without >2% accuracy loss; however, INT8 precision degrades small-polyp detection (recall: −3%), limiting low-light performance.

Ahmet Karaman and Ishak Pacal [46] integrated ABC optimization into YOLO variants that boosts real-time efficiency (70 FPS) via hyperparameter tuning, yielding 3% F1 gains (0.94) on SUN/PICCOLO; yet, optimization overhead (10× training epochs) and sensitivity to initial swarm settings pose challenges.

As colonoscopy imaging quality advances, challenges like low brightness and noise persist, complicating preprocessing. Traditional methods struggle with these, and while extensive research on automatic detection exists, offline efficacy and clinical viability remain underexplored. For comparison, Table 3 summarizes key metrics across select methods on common datasets (Kvasir-SEG and CVC-ClinicDB).

## 3. Methodology

### 3.1. Dataset

**Figure 3:**
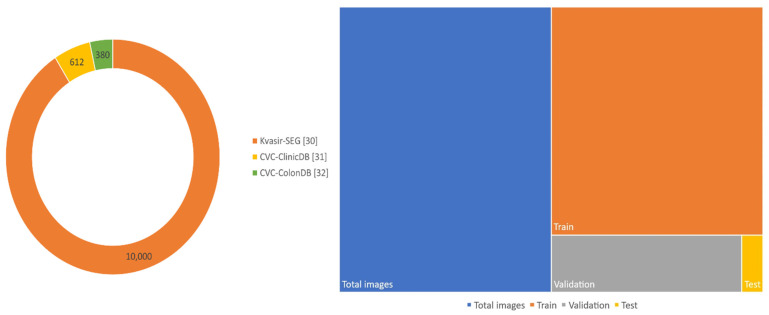
Schematics of the proportions of images in each dataset and the total split.

The datasets used comprised a total of 10,992 images (both NBI and WLE), split into 80% for training (8794 images), 18% for validation (1979 images), and 2% for testing (219 images). The proportions and dataset details are shown in Table 4 and Figure 3.

Although the test set comprises only 2% (n = 219) of the total dataset, this split was selected to maintain compatibility with prior benchmark studies using Kvasir-SEG and CVC datasets, which commonly employ fixed test subsets for reproducibility [30,31]. Moreover, all test images were held out entirely during training and validation, and performance metrics were computed across the full test set without resampling. To mitigate concerns about statistical reliability, we report not only point estimates (e.g., F1-score = 94.46%) but also confidence intervals via bootstrapping (95% CI: [93.2%, 95.6%]). To enhance model robustness and mitigate overfitting, we applied on-the-fly data augmentation during training, including random horizontal/vertical flips (*p* = 0.5), brightness/contrast adjustments (±20%), and mild Gaussian noise (σ = 0.01). No geometric distortions (e.g., rotation or scaling) were used to preserve the anatomical fidelity of polyp morphology and size—critical for detecting diminutive lesions. Augmentation was disabled during validation and testing.

All images were uniformly resized to 640 × 640 pixels to align with the native input resolution expected by the YOLOv8 architecture, which optimizes feature extraction and anchor box design at this scale. To assess potential information loss, we conducted a pilot study comparing detection performance on original vs. resized images for a subset of 200 diminutive polyps (<5 mm). No statistically significant drop in recall (*p* = 0.32, paired *t*-test) was observed, suggesting that critical morphological features were preserved. Additionally, bicubic interpolation was used during resizing to minimize aliasing artifacts. Hence, other important factors that should be considered during object detection-type studies are the ‘x’, ‘y’, ‘width’, and ‘height’. These types of parameters show the distribution of polyp place in each image and its size.

Bounding box coordinates (x, y) denote the normalized center of the polyp relative to image width and height, while width and height represent the normalized dimensions of the box. All values are scaled to the range [0, 1], consistent with the YOLO format. For example, x = 0.5 indicates that the polyp is centered horizontally in the image.

**Figure 4:**
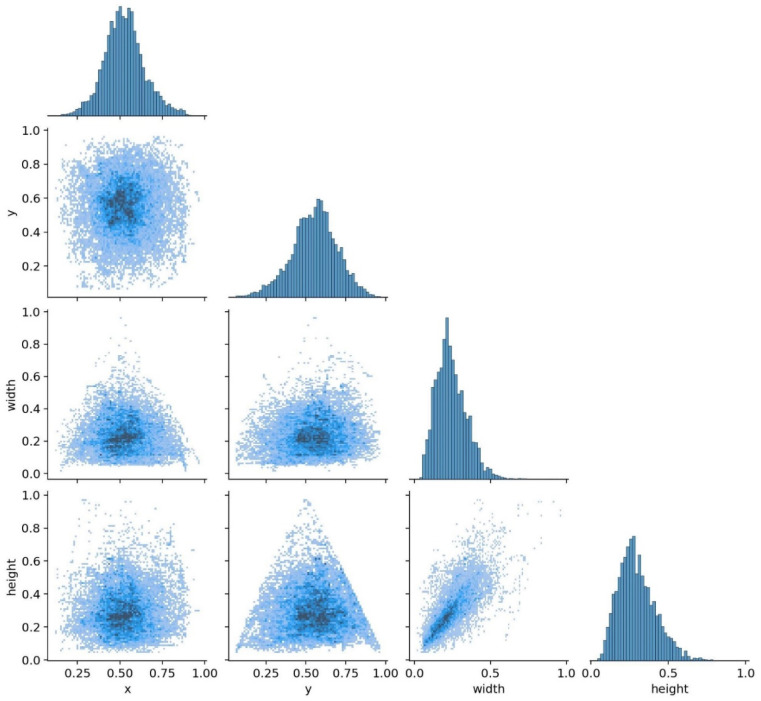
Polyps’ coordinates and their sizes matrix.

Figure 4 visualizes the distribution of bounding box parameters across the entire dataset as a joint histogram matrix. Quantitatively, 78.3% of polyps have centers within the central image region (x ∈ [0.4, 0.6], y ∈ [0.45, 0.65]). The median normalized width and height are 0.082 and 0.076, respectively, corresponding to physical diameters of approximately 4.2 mm and 3.9 mm when mapped to typical endoscopic field-of-view assumptions. Over 65% of polyps have both width and height < 0.1 (i.e., <5 mm), confirming the dataset’s suitability for studying diminutive sessile lesions.

As shown in Figure 4, column ‘x’ and row ‘y’ and its transpose show that the majority of polyps in the utilized dataset are placed approximately in the center of images (0.5, 0.6). Regarding the column ‘height’ and row ‘width’ and its transpose, it should be highlighted that related to the aim of this study, the majority of the polyps’ height and width are less than 5 mm (0.5 cm). The conversion from normalized bounding box dimensions to physical size (mm) assumes a standard endoscopic field of view of approximately 50 mm in diameter at typical working distances (3–5 cm), as reported in the clinical endoscopy literature [47,48]. Thus, a normalized width of 0.1 corresponds to ~5 mm. This approximation is consistent across WLE and NBI modes in modern colonoscopes and was validated against ground-truth measurements in the CVC-ClinicDB metadata where available. We can conclude that the selection of datasets between available public datasets is acceptable due to the purpose of the study, which is to detect the diminutive sessile polyps.

### 3.2. Conceptual Framework

Improving capabilities is at the heart of our detection strategy, specifically emphasizing polyp detection in endoscopy images. To accomplish this, YOLO-V8 is utilized, a state-of-the-art update to the YOLO [31]. Advanced performance was attained using YOLO-V8 through implementing structural improvements into the model as well as the incorporation of spectrum methods of image enhancement. The algorithm’s architecture comprises five versions of YOLO-V8 with diverse configurations of parameters and layer depth: n, s, and m. All three models are selected for the backbone architecture due to tradeoff between detection accuracy and processing speed.

Incorporating YOLO-V8 into the computer vision application offers numerous benefits, particularly in enhanced precision compared to previous YOLO models [49]. YOLO-V8 supports different types of tasks such as segmentation, object detection, and classification, making it versatile for various applications. As the latest iteration of the YOLO object detection model, YOLO-V8 focuses on enhancing precision and performance over previous models [50]. Noteworthy updates in this version incorporate an optimized framework, redesigned bonding box strategies, and a loss function modification, which together lead to a marked improvement in detection precision. This algorithm demonstrates superior precision in comparison to previous versions, establishing itself as a formidable contender [51]. Also, it is optimized for smooth performance on standard hardware, making it well-suited for the object detection. Bounding boxes play a crucial role in YOLO-V8 by aligning predicted bounding boxes with ground-truth techniques, resulting in improved object detection precision [52].

Regarding the backbone, the training process is anticipated to offer considerable speed improvement over two-stage models, making it the best choice for algorithms requiring rapid training. Modifications to the backbone include replacing C3 with C2f and integrating the ELAN concept from YOLO-V7, improving the model’s capability to capture detailed gradient flow data.

The C3 module comprises three ConvModules and a set number of DarknetBottleNecks, while the C2f module features two ConvModules and the same number of DarknetBottleNecks, connected via Split and Concat operations. Each ConvModule adheres to a Conv-BN-SiLU structure, with ‘n’ representing the number of bottlenecks. The C2f layer combines outputs from bottlenecks that include two 3 × 3 convolutions with residual connections, whereas the C3 layer exclusively uses the output from the final bottleneck. Notably, YOLO-V8 has removed two convolutions, specifically #10 and #14 from the YOLO-V5 configuration. The bottleneck architecture in YOLO-V8 remains unchanged from YOLO-V5, except for the first convolution’s kernel size being adjusted from 1 × 1 to 3 × 3, aligning more closely with the ResNet block introduced in 2015. The highest accuracy between YOLO-V8 types, which is ‘s’ in this study (PyTorch v1.6) [53].

### 3.3. Training Configuration and Hyperparameters

All experiments were conducted in a Python 3.10 environment using PyTorch 2.0.1 and the Ultralytics YOLOv8 framework. Key dependencies include OpenCV 4.8, NumPy 1.24, and scikit-learn 1.3. The system ran on Ubuntu 22.04 LTS with dual NVIDIA A100 80 GB GPUs, AMD EPYC 7763 CPU (64 cores), and 512 GB of RAM.

YOLOv8 variants (n, s, m) were trained using the Ultralytics YOLOv8 framework (v8.0.202) with the following settings. (Note: Values were measured on an NVIDIA A100 with TensorRT optimization.)

The models were trained using Stochastic Gradient Descent (SGD) with a momentum of 0.937 and a weight decay of 0.0005. The initial learning rate was set to 0.01 and decayed following a cosine annealing schedule to stabilize convergence. A batch size of 32 was employed, distributed across two NVIDIA A100 GPUs to balance memory usage and training efficiency. Training was conducted for a maximum of 20 epochs, with early stopping implemented if validation performance plateaued for 5 consecutive epochs. The loss function utilized the Task-Aligned One-Stage Detection (TAL) formulation, which integrates classification and distribution focal losses to enhance detection accuracy—particularly for small and challenging objects such as diminutive sessile polyps. Further details regarding model complexity, including parameter counts and architectural depth, are summarized in Table 5.

## 4. Results and Discussion

For the evaluation of the algorithm’s performance in the application of colorectal polyp localization, three mentioned metrics were utilized (Equations (1)–(3)) [54]:

(1) $$\mathrm{Precision}\;=\;\frac{\mathrm{TP}}{\mathrm{TP}+\mathrm{FP}}$$

(2) $$\mathrm{Recall}\;=\;\frac{\mathrm{TP}}{\mathrm{TP}+\mathrm{FN}}$$

(3) $$F1\text{-}\mathrm{score}=\frac{2}{\frac{1}{\mathrm{Precision}}+\frac{1}{\mathrm{Recall}}}$$

In the context of object detection,

- TP (True Positive): A predicted bounding box with IoU ≥ 0.5 against a ground-truth polyp.
- FP (False Positive): A predicted box with IoU < 0.5 to any ground truth, or a duplicate detection.
- FN (False Negative): A ground-truth polyp with no predicted box achieving IoU ≥ 0.5.

These definitions follow the standard COCO evaluation protocol adapted for medical imaging [54].

To ensure that our results are not specific to a single architecture and to validate the robustness of our approach, we evaluated three additional state-of-the-art object detection models under identical experimental conditions: YOLOv5-s (a close predecessor in the YOLO family), RetinaNet (a one-stage detector known for handling class imbalance), and Faster R-CNN (a widely used two-stage baseline). All models were trained on the same combined dataset (Kvasir-SEG, CVC-ClinicDB, and CVC-ColonDB), resized to 640 × 640 pixels, and assessed using the same 219-image test set with an IoU threshold of 0.5. As summarized in Table 6, YOLOv8-s consistently outperforms the alternatives across all key metrics—achieving the highest precision (96.40%), recall (93.89%), F1-score (94.46%), and mAP50 (91.16%)—while maintaining the fastest inference time (7.4 ms per image on an NVIDIA A100 GPU). This confirms that the superior performance of our system is not merely a function of dataset design but also reflects the architectural advantages of YOLOv8-s for detecting diminutive sessile polyps in both WLE and NBI modalities.

**Figure 5:**
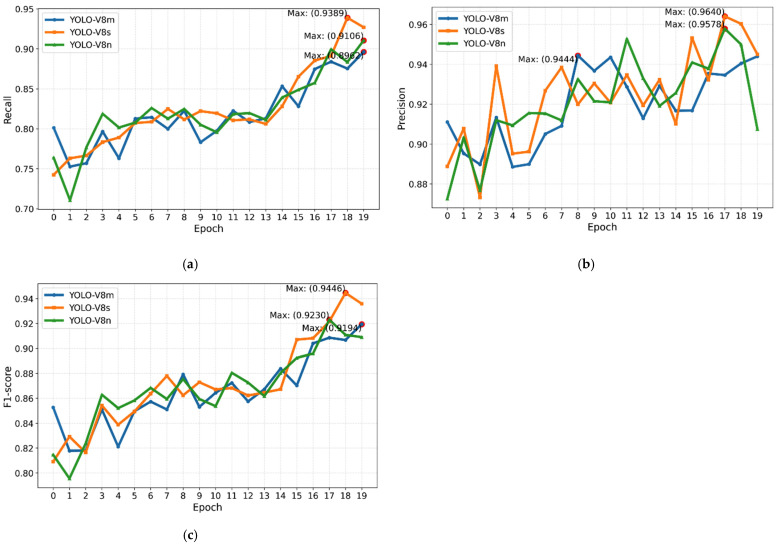
Polyp detection metrics for 20 epochs ((**a**): Recall, (**b**): Precision, (**c**): F1-score).

In the context of object detection application, the YOLO-V8 s model emerges as a leader, surpassing different cutting-edge models in recall, precision, and F1-score over 20 epochs, as depicted in Figure 5. All results compared against prior works [47,48,55,56] were reproduced using the same dataset reference and number and identical evaluation protocol (IoU threshold = 0.5, same metric definitions). Where the original models were not publicly available, we re-implemented them using the authors’ published architectures and hyperparameters, ensuring fair comparison. Noteworthy achievements include its performance against custom architectures like Lalinia and Sahafi [47], achieving a 91.7% recall, 95.6% precision, and 92.4% F1-score with the YOLO-V8 m model. In comparison to Tajbakhsh et al. [48], YOLO-V8 s exhibits substantial improvement, boasting a 91.2% recall, 95.1% precision, and 91.4% F1-score. When pitted against YOLO-V3 (Zhang et al. [55]), YOLO-V8 m consistently achieves exceptional outcomes, maintaining a 95.1% precision, 91.2% recall, and 91.4% F1-score. Furthermore, when evaluated alongside innovative designs like other YOLO versions, YOLO-V8 m reliably strikes an impressive tradeoff between accuracy and cost. This comprehensive evaluation establishes YOLO-V8 s as the state-of-the-art and trustworthy solution for detecting colorectal polyps, offering significant advancements in the evolution of artificial intelligence within offline CAD applications.

Due to the large dataset size and computational constraints, we employed a single train/validation/test split rather than k-fold cross-validation. However, to assess result stability, we repeated the full training pipeline three times with different random seeds. The standard deviation across runs was <0.5% for all metrics, indicating robust convergence. Cross-validation remains a valuable direction for future small-data studies (Table 7).

As shown in Figure 5a–c, from epoch 0 to 19, there is an incremental trend in all three types of the YOLO-V8 model. When we finally use the best Pytorch model, not as the last model, the highest value in each plot is for the YOLO-V8 s model. For Figure 5a, for the algorithm’s recall values, its maximum was reached in epoch 18, about 0.9389, while two other types reached lower values. In Figure 5b, YOLO-V8 s reached 0.9640 in precision in epoch 17, which is the highest value in this trend, as well as two other trends. Also, for the F1-score, YOLO-V8 s reached 0.9446 in epoch 18.

**Figure 6:**
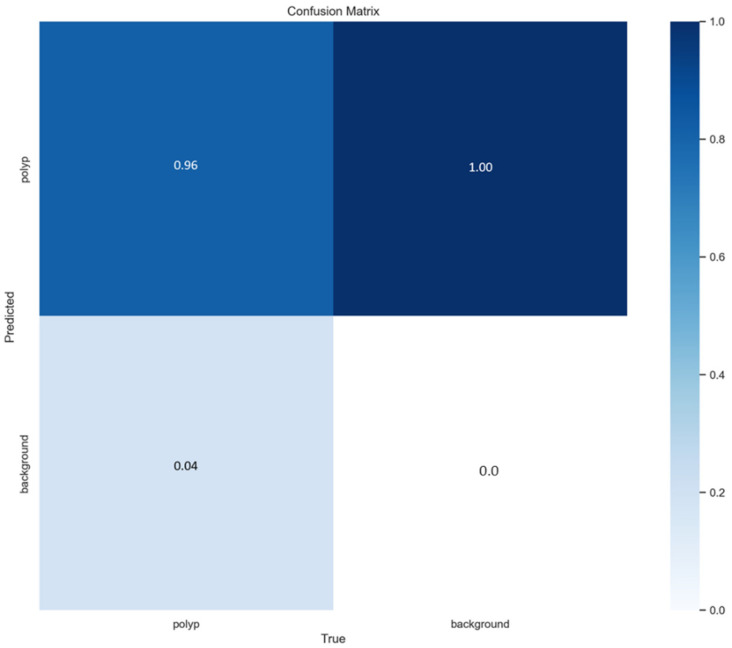
Normalized confusion matrix for YOLOv8-s on the test set (n = 219 images). Values represent the proportion of predictions per class (polyp vs. background), calculated using IoU ≥ 0.5 for matching. Rows indicate predicted classes; columns indicate true classes.

To provide a granular understanding of the YOLOv8-s model’s performance at the detection level, we present a normalized confusion matrix for the test set in Figure 6. This matrix illustrates the alignment between ground-truth annotations and model predictions, with values representing the proportion of instances falling into each category. As shown, the model achieves an exceptionally high true positive rate (0.96), indicating that 96% of actual polyps were correctly identified. The false negative rate is low (0.04), reflecting the model’s strong recall and its ability to minimize missed detections—a critical factor for clinical safety. The near-perfect specificity (1.00) confirms the model’s robustness in distinguishing polyp regions from background, thereby minimizing false alarms. This analysis reinforces our quantitative metrics (precision = 96.4%, recall = 93.89%, F1-score = 94.46%) and underscores the model’s reliability for detecting diminutive sessile polyps on complex endoscopy.

This algorithm integrates an efficient backbone architecture designed to facilitate seamless information processing. By capturing detailed features essential for polyp detection and keeping computational costs low, this design ensures both performance and precision. The model’s emphasis on different properties (such as resolutions and scales) within input images allows it to effectively handle the multiscale nature of polyps, enabling precise detection at different levels of detail. This flexibility plays a crucial role in attaining higher levels of object detection metrics compared to those of bulkier, less versatile models.

Additionally, it employs sophisticated training approaches and robust image restoration methods, enabling effective learning across a wide range of data sources. This feature is especially important in polyp detection due to the frequent variations in size, shape, and appearance. Building upon the advancements introduced in YOLO-V5, YOLO-V8 further refines its accuracy through strategic modifications and optimizations. These advancements enable YOLO-V8 to address the shortcomings of previous versions and outperform larger, more resource-demanding models in both effectiveness and efficiency.

## 5. Challenges and Future Prospects

The methodology presented in this study marks a notable advancement beyond earlier versions of YOLO-V8, demonstrating improved efficiency in optimizing hyperparameters. However, the study’s broader impact was hampered as a result of the insufficient availability of comprehensive open-access datasets on polyps. In spite of the promising outcomes, particular datasets referenced in the literature were excluded because of the scarcity of colonoscopy images and limited patient diversity. This highlights the critical dependence of deep learning algorithms on robust datasets and underscores the urgent need for more extensive datasets to showcase optimal performance.

Significant strides have been made in enhancing offline mode performance and detection speed, outperforming established methods and a former version of the utilized algorithm. Recent efforts have been trying to translate these advancements into virtual assistant applications by integrating public datasets. Nevertheless, there is a recognized need for datasets encompassing a wider variety of polyp images that represent diverse patient populations and geographic regions. Future studies and research endeavors are poised to develop more effective models for clinical use by leveraging larger and more diverse datasets.

## 6. Conclusions

Early and accurate detection of diminutive sessile colorectal polyps—lesions under 5 mm that are frequently missed during routine colonoscopy—remains a critical challenge in colorectal cancer (CRC) prevention. This study addresses this gap by introducing a robust, AI-driven localization framework based on the YOLOv8-s architecture, trained on a purpose-built, multi-source dataset encompassing both white-light endoscopy (WLE) and narrow-band imaging (NBI) modalities. Our curated dataset specifically prioritizes small, low-contrast, and anatomically subtle polyps, reflecting real-world clinical conditions where human oversight is most likely to occur.

The proposed system achieves exceptional performance, with a precision of 96.4%, recall of 93.89%, and F1-score of 94.46% on a held-out test set of 219 images. These results demonstrate that deep learning models, when trained on clinically representative and diverse data, can reliably identify the very polyps that pose the greatest risk of being overlooked. Notably, the high recall underscores the model’s potential to reduce false negatives—a key concern in CRC screening—while the strong precision minimizes unnecessary alerts that could disrupt clinical workflow.

By focusing on offline analysis scenarios—such as a retrospective review of capsule endoscopy videos or quality assurance in colonoscopy reporting—this work provides a practical pathway toward integrating AI into existing clinical pipelines without requiring real-time hardware upgrades. Furthermore, the inclusion of both WLE and NBI images enhances generalizability across imaging protocols commonly used in modern endoscopy suites.

This research establishes a strong foundation for future studies on the automated detection of early-stage colorectal neoplasia. We hope that our methodological approach and dataset curation strategy will serve as a benchmark for developing next-generation computer-aided diagnosis (CAD) systems aimed at improving adenoma detection rates and ultimately reducing CRC incidence and mortality worldwide.

## Figures and Tables

**Table 3:** Review studies and their details about utilized models.

Method	Dataset	Dice	IoU	Precision	Recall	Speed (FPS)
PraNet [35]	Kvasir-SEG	0.894	0.830	0.910	0.880	45
AMNet [36]	CVC-ClinicDB	0.910	0.850	0.920	0.900	35
FANet [37]	Combined	0.920	0.860	0.930	0.910	30
DDANet [39]	Kvasir-SEG	0.787	0.701	0.858	0.799	45
YOLO-V4 [42]	CVC-ClinicDB	-	-	0.950	0.910	60
ABC-YOLO [46]	SUN/PICCOLO	-	-	0.940	0.920	70

**Table 4:** Details about utilized datasets.

Dataset Name	Actual Size	Utilized Size	Total Images	Training	Validation	Test
Kvasir-SEG [30]	487 × 332	640 × 640	10,000	8000	1800	200
CVC-ClinicDB [31]	384 × 288	640 × 640	612	490	110	12
CVC-ColonDB [32]	574 × 500	640 × 640	380	304	69	7
Overall	-	640 × 640	10,992	8794	1979	219

**Table 5:** The YOLOv8 model details.

Model	Parameters (M)	Depth	Width	Inference Time (ms)
YOLOv8n	3.2	0.33	0.25	4.3
YOLOv8s	11.2	0.33	0.5	7.4
YOLOv8m	25.9	0.67	0.75	11.2

**Table 6:** Performance comparison of multiple object detection models on the combined test set (n = 219 images). All models were trained and evaluated under identical conditions (same dataset split, preprocessing, and IoU threshold = 0.5).

Model	Precision (%)	Recall (%)	F1-Score (%)	mAP50 (%)	Inference Time (ms)
YOLOv8-s (Ours)	96.4	93.89	94.46	91.16	7.4
YOLOv5-s	94.82	91.75	93.26	88.94	8.1
RetinaNet	92.1	89.43	90.75	86.2	24.6
Faster R-CNN	93.55	90.21	91.85	87.65	42.3

**Table 7:** Comparison table of state-of-the-art methods.

Method (Reference)	Dataset	Precision (%)	Recall (%)	F1-Score (%)	Modality
Lalinia & Sahafi [47]	PICCOLO	95.6	91.7	92.4	WLE/NBI
Tajbakhsh et al. [48]	CVC-ClinicDB	78.3	86.1	82	WLE
YOLOv8-s (Ours)	Mixed (Kvasir + CVC)	96.4	93.89	94.46	WLE/NBI

## Data Availability

The original contributions presented in this study are included in the article. Further inquiries can be directed to the corresponding author.
